# Positioning of NORs and NOR-bearing chromosomes in relation to nucleoli

**DOI:** 10.1016/j.jsb.2007.06.012

**Published:** 2007-10

**Authors:** Markéta Kalmárová, Evgeny Smirnov, Martin Mašata, Karel Koberna, Anna Ligasová, Alexey Popov, Ivan Raška

**Affiliations:** Institute of Cellular Biology and Pathology, First Faculty of Medicine, Charles University in Prague, Czech Republic; Department of Cell Biology, Institute of Physiology, Academy of Sciences of the Czech Republic, v.v.i., Albertov 4, 128 01 Prague 2, Czech Republic

**Keywords:** Chromosome territories, NORs, Nucleoli, rDNA, Transcription activity

## Abstract

It is widely accepted that chromosomes occupy more or less fixed positions in mammalian interphase nucleus. However, relation between large-scale order of chromosome positioning and gene activity remains unclear. We used the model of the human ribosomal genes to address specific aspects of this problem. Ribosomal genes are organized at particular chromosomal sites in clusters termed nucleolus organizer regions (NORs). Only some NORs, called competent are generally accepted to be transcriptionally active during interphase. Importantly in this respect, the regularities in distribution of competent, and non-competent NORs among the specific chromosomes were already established in two human-derived cell lines: transformed HeLa and primary LEP cells. In the present study, using FISH and immunocytochemistry, we found that in HeLa and LEP cells the large-scale positioning of the NOR-bearing chromosomes with regard to nucleoli is linked to the transcription activity of rDNA. Namely, the tendency of rDNA-bearing chromosomes to associate with nucleoli correlates with the number of transcriptionally competent NORs in the respective chromosome homologs. Regarding the position of NORs, we found that not only competent but also most of the non-competent NORs are included in the nucleoli. Some intranucleolar NORs (supposedly non-competent) are situated on elongated chromatin protrusions connecting nucleoli with respective chromosome territories spatially distanced from nucleoli.

## 1 Introduction

During interphase, chromosomes exist in the form of well discernible, though highly porous, territories (Cremer et al., 1993; Cremer and Cremer, 2001, 2006; Foster and Bridger, 2005; Parada and Misteli, 2002; Pederson, 2004; Verschure et al., 1999; Visser et al., 2000). It is widely accepted that these chromosome domains occupy more or less fixed positions in the mammalian interphase nucleus, depending on the gene density, replication timing and the size of chromosome territory (Manders et al., 1999; Parada et al., 2003; Sun et al., 2000; Vazquez et al., 2001; Walter et al., 2003; Zink et al., 1998). Results of several studies indicate that the location of chromosomes is related to the activity of their genes (Gilbert and Ramsahoye, 2005; Mahy et al., 2002). The gene-rich chromosomes are preferentially located at the nuclear center, though the preference disappeared after inhibition of transcription, suggesting that the chromosome positioning may depend on the transcription activity. Other data argue that particular chromosome positioning places genes into special neighborhood favorable for their expression or silencing (Parada and Misteli, 2002). The situation is complicated by recent data indicating that chromosome territories partially intermingle in human cell nuclei (Branco and Pombo, 2007) and some genes can be found far beyond the area defined as chromosome territory (Bártová et al., 2007; Baxter et al., 2002; Chubb and Bickmore, 2003; Kioussis, 2005; Mahy et al., 2002; Morey et al., 2007; Wegel and Shaw, 2005). Thus, the large-scale order of chromosome positioning and its relation to gene activity remain unclear.

Human ribosomal genes represent a convenient model to address specific aspects of this problem. These genes form clusters, named nucleolus organizer regions or NORs, in each of the acrocentric chromosomes 13, 14, 15, 21 and 22 (Henderson et al., 1972; Long and Dawid, 1980; Puvion-Dutilleul et al., 1991). Essential components of the RNA polymerase I transcription machinery, including Upstream Binding Factor (UBF), can be detected by immunocytochemistry or silver staining on some NORs, termed “transcriptionally competent” or “competent”, during mitosis. It is generally accepted that competent NORs are transcriptionally active during previous interphase (Gebrane-Younes et al., 1997; Roussel et al., 1996; Weisenberger and Scheer, 1995). Nucleoli reform after mitosis around transcriptionally active, and therefore necessarily competent, NORs (Benavente et al., 1987; Jimenez-Garcia et al., 1994; Ochs et al., 1985), and the integrity of nucleoli depends on expression of ribosomal genes (Dousset et al., 2000; Melese and Xue, 1995; Scheer and Hock, 1999). However, the position of the non-competent NORs that exhibit a condensed chromatin structure (O’Sullivan et al., 2002), and chromosomes carrying the non-competent NORs, with respect to nucleoli remains unclear.

Importantly, we have already shown regularities in distribution of competent as well as non-competent NORs among the specific chromosomes in two human-derived cell lines, transformed HeLa and primary LEP cells (Smirnov et al., 2006), and established that all HeLa cells, and more than 95% LEP cells, contain at least one non-competent NOR (Smirnov, unpublished observations). More specifically, we showed that in the aneuploid HeLa cells the NORs belong to acrocentric chromosomes 13, 14, 15, 21, 22 and a metacentric one painted with probe for chromosome 15. The signals of transcriptional competence (silver or UBF signals) were usually present on one chromosome 13, one chromosome 14, all chromosomes 15 (three acrocentrics and one metacentric), two chromosomes 21, and two chromosomes 22. In LEP cells, 10 NORs were regularly observed on mitotic chromosome spreads. In these cells, both copies of the chromosome 15, but only one of the chromosomes 13, regularly contained transcriptionally competent NORs. Both chromosomes 14 contained competent NORs in 85% cases. Small acrocentrics 21 and 22 displayed less regularity: one or both homologs carried competent NORs with comparable frequencies (Smirnov et al., 2006).

In the present study, we expand the results of Smirnov et al. (2006) and analyze nuclear positions of competent and non-competent NORs, as well as chromosomes bearing NORs, with respect to nucleoli in HeLa and LEP interphase cells.

## 2 Materials and methods

### 2.1 Cell culture

HeLa, aneuploid cell line, that have stable karyotype without considerable variations (Macville et al., 1999; Smirnov et al., 2006), and primary LEP cells were cultivated in flasks or on coverslips at 37 °C in Dulbecco’s modified Eagle’s medium (DMEM, Sigma, USA) containing 10% fetal calf serum, 1% glutamine, 0.1% gentamycin, and 0.85 g/l NaHCO_3_ in atmosphere supplemented with 5% CO_2_.

### 2.2 Antibodies and DNA probes

Commercial Cy3- and FITC-labeled whole chromosome painting probes for human chromosomes 13, 14, 15, 21 and 22, supplied ready to use in hybridization mixture (Appligene Oncor, USA), and pA and pB rDNA probes, prepared from a pA and pB plasmid constructs (Erickson et al., 1981), kindly donated by James Sylvester (Nemours Children’s Clinic Research, Orlando, FL) were used. The pA probe contains the 3′ end of 18S rDNA, the 5.8S rDNA, both internal transcribed spacers, and most of the 28S rDNA. The pB probe contains the promoter, the external transcribed spacer, and the 5′ end of the 18S subfragment. The probes were labeled by biotin using nick-translation kit BIONICK Labeling System (Gibco-BRL, Invitrogen) according to the manufacturer’s instructions. The rDNA probes were stored in hybridization mixture containing 25 ng of probe, 0.5 mg/ml sonicated salmon sperm DNA, 50% deionized formamide, 2× SSC and 10% dextran sulfate at −20 °C. Both rDNA probes exhibited the same pattern of FISH-labeling. Therefore, only the results obtained with pB probe were used for statistical analysis.

Primary monoclonal antibody against mouse fibrillarin (clone 17C12), kindly donated by Kenneth M. Pollard (Scripps Research Institute, La Jolla, CA), was used for immunovisualization of nucleoli. Biotinylated rDNA probe was labeled after FISH with monoclonal rabbit anti-biotin antibodies (Enzo, Roche). Secondary anti-mouse and anti-rabbit antibodies (Jackson ImmunoResearch Laboratories) were conjugated with Cy3 or FITC.

### 2.3 Immunofluorescence

Cells growing on coverslips were washed in phosphate-buffered saline (PBS), fixed in methanol at −20 °C for 30 min and air-dried. Following three washes in PBS, the cells were incubated with anti-fibrillarin antibody, washed in PBS and incubated with secondary antibodies conjugated with either FITC or Cy3.

### 2.4 Immuno-FISH and FISH

The combined detection of fibrillarin and *in situ* hybridization (immuno-FISH) was performed after Pliss et al., 2005. After fibrillarin immunolabeling, as described above, the cells were postfixed with methanol/acetic acid (3:1) overnight at −20 °C, then the regular FISH procedure followed (Pliss et al., 2005), except the post hybridization washing. Namely, the cells were washed in 50% formamide in 2× SSC, pH 7, for 15 min at 43 °C, in 0.1% Tween 20/2× SSC for 8 min at 43 °C; in 0.1% Igepal (ICN Biomedicals, Inc.)/4× SSC for 3 × 4 min at 37 °C, in PBS 3 × 3 min at RT (Harničarová et al., 2006). After FISH, biotinylated rDNA probes were detected using respective primary and secondary antibodies (Section 2.2).

For the combined detection of fibrillarin and double-FISH (i.e. triple labeling), the fibrillarin immunolabeled cells were first photographed and their position on the slide marked before methanol–acetic acid postfixation. Then the FISH with rDNA and chromosome probes were performed, and the same cells were photographed again. This method was used to achieve the best visualization of nucleoli.

To ensure the detection of all extranucleolar rDNA foci, we employed an alternative approach avoiding fibrillarin labeling. Accordingly, the cells were fixed in methanol/acetic acid (3:1) for 30 min at −20 °C. After air-drying the cells were processed for FISH as described above, and nucleoli were visualized by phase contrast and as DAPI negative areas. Although the nucleolar areas could not be identified as precisely as after fibrillarin immunolabeling, the numbers of the extranucleolar rDNA foci matched well with the results obtained by the immuno-FISH. Thus we observed in HeLa cells no extranucleolar foci in 68% cells, one focus in 20% cells, two foci in 5% cells, three foci in 4% cells and four foci in 1% cells (compare with Fig. 4b).

The results of all single labeling (fibrillarin immunolabeling and FISH), double labeling (fibrillarin immunolabeling combined with FISH and double-FISH) and triple labeling experiments (fibrillarin immunolabeling and double-FISH) were compatible.

Coverslips were mounted in Mowiol supplemented with DABCO and viewed using Olympus AX70 Provis equipped with the Photometrics CCD camera or Leica TCS NT confocal microscope.

All statistical evaluations were obtained by analysis of 100 HeLa and LEP cells.

### 2.5 Mathematical 2D random model system

We chose 2D-analysis, because it allows statistical evaluation of large numbers of images. 2D-analysis has been used for the study of nuclear positioning of DNA loci and chromosome territories in cells that are grown on glass surface and have flattened nuclei (see e.g. Mahy et al., 2002; Parada et al., 2004; Taslerová et al., 2006; Volpi et al., 2000), and similar results with respect to the mutual orientation of these objects were obtained by 2D- and 3D-analysis (Mahy et al., 2002; Morey et al., 2007).

We used a model in which polygonal chromosomes were randomly positioned within elliptic nucleus containing randomly positioned round nucleoli. The parameters: area of the nucleus, its major axis length, total area occupied by nucleoli and chromosomes, the number of nucleoli and chromosomes were obtained as mean values of measurements and counts on the cells after hybridization. The geometric parameters were measured by means of the Soft Imaging System (Analysis program).

## 3 Results

### 3.1 Nucleolar association of the interphase NOR-bearing chromosomes correlates with transcriptional competence of their NORs in HeLa and LEP cells

**Fig. 1:**
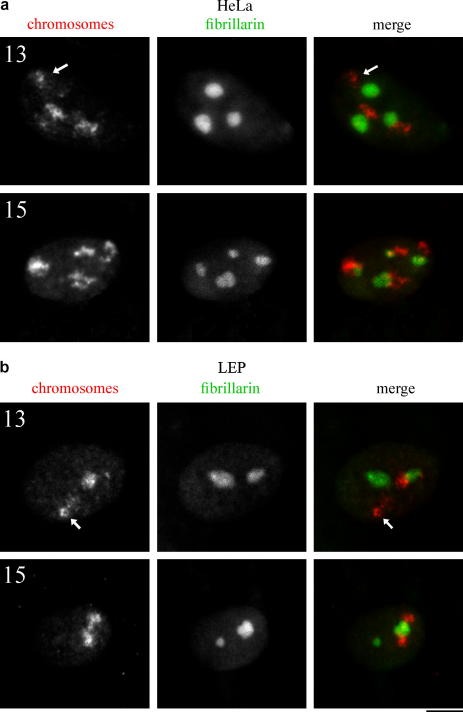
Association of the chromosomes 13 and 15 with nucleoli in interphase HeLa (a) and LEP (b) cells. FISH signal with the specific chromosome probes (in red) was observed in interphase cells. Immunocytochemistry with fibrillarin was used to visualize nucleoli (in green). The transformed HeLa cells contain three homologs of the chromosomes 13 and four homologs of the chromosome 15. The primary LEP cells have normal karyotype with two chromosomes 13 and 15. In both HeLa and LEP cells, the chromosome homologs 15 appeared usually in close association with nucleoli, while one of the homologs 13 was often distanced from the nucleoli (arrow). Bar: 10 μm.

**Fig. 2:**
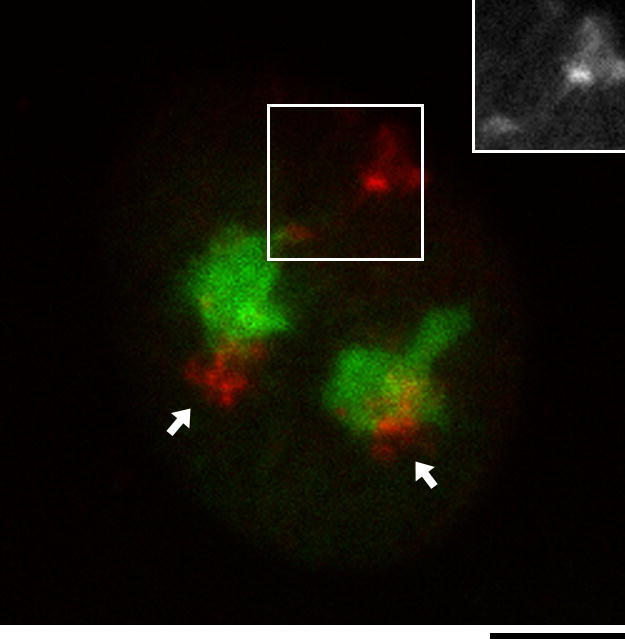
A thin protrusion between the extranucleolar chromosome territory and nucleolus. Two chromosome homologs 14 (in red; arrows) in the HeLa cell are closely associated with fibrillarin-positive nucleoli (in green). The third chromosome homolog 14 (in red) is distanced from nucleolus and connected to it via a long chromatin protrusion that ends with a thickening at the contact point with the nucleolus. Since the corresponding chromatin signal is weak in the merge image, this protrusion is shown in the one channel grey scale insert. Bar: 10 μm.

We analyzed nucleolar association of the NOR-bearing chromosomes in HeLa (containing in average 4.03 ± 0.12 nucleoli; mean value and standard error are indicated) and LEP cells (containing in average 2.04 ± 0.10 nucleoli), bearing in mind the established occurrence of competent and non-competent NORs in homologous NOR-bearing chromosomes (Smirnov et al., 2006). Fluorescent *in situ* hybridization with probes for chromosomes 13, 14, 15, 21 and 22 was performed on interphase cells (Fig. 1). Some of the studied chromosomes had no significant contact with nucleolus revealed by fibrillarin immunolabeling that is commonly used for the visualization of nucleoli. Such “extranucleolar” chromosomes were frequently distanced from nucleoli by more than one micrometer. On the other hand, the majority of NOR-bearing chromosome territories were associated with nucleoli. Different forms of the nucleolar association were observed for different chromosome homologs (Fig. 1). In the case of chromosomes 13 and 14 in both HeLa and LEP cells, the painted part of chromosome was typically straight, more or less elongated and entering nucleolus at one point. Second form is represented by the chromosomes 15 which, in both HeLa and LEP cells, often penetrated to the centre of nucleolus or even traversed its area. Other chromosomes (especially the chromosomes 21 and 22 in HeLa cells) appeared as semilunar structures embracing nucleolus. All these cases were considered here as an association of chromosome with nucleolus. Sometimes, we observed long thin filaments connecting the extranucleolar chromosome territory with nucleolus (Fig. 2; see also Section 3.2). These cases were not considered as nucleolar associations.

**Fig. 3:**
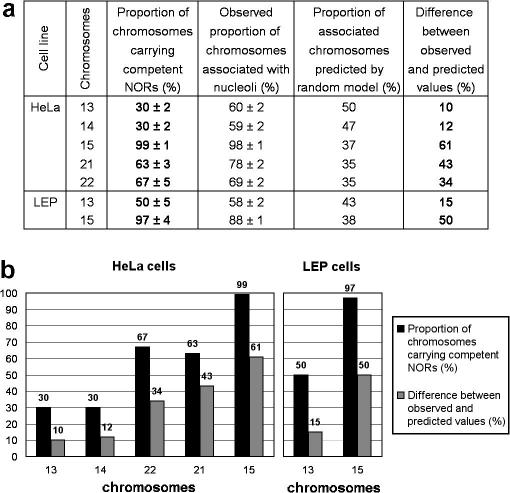
Transcription competence of the NORs belonging to different chromosomes correlates with nucleolar associations of these chromosomes. (a) The percentage of the nucleoli closely associated chromosomes was counted in HeLa and LEP cells. Respective numbers of the competent NORs in different NOR-bearing chromosomes in these cells have been obtained from colocalization experiments (Smirnov et al., 2006). The results are compared with the data provided by mathematical random 2D model where the percentage of the association depends on the size of chromosomes detected by chromosomal probe. In all studied cases the differences between the observed data and those predicted by the model significantly exceed the level of measurement error and reflect the affinity of certain NOR-bearing chromosome homologs towards nucleoli. Mean values and standard errors are indicated. (b) The relevant bar diagrams are shown to clearly document the observed positive correlation between the proportion of chromosomes carrying competent NORs (black bars corresponding to the third column in Fig. 3a) and the close nucleolar association of the chromosomes (gray bars corresponding to the last column in Fig. 3a). Accordingly, the chromosomes in HeLa cells are, in contrast to Fig. 3a, arranged upon the increasing value of their nucleolar association.

The percentage of the chromosomes associated with nucleoli in HeLa and LEP cells is shown in Fig. 3. In this Fig. we also compared the experimental data with the results provided by a mathematical 2D random model system in which chromosomes and nucleoli were randomly scattered within an elliptic nucleus (see Section 2). In this model nucleolar association depends on the chromosomal size which was determined by hybridization signal. Thus, the difference between the observed data and those predicted by the model reflected the affinity of a certain chromosome type towards nucleoli and this affinity was not influenced by the size of chromosomes. In all studied cases this difference significantly exceeded the level of measurement error, and was proportional to the value (percentage) of the nucleolar association for each type of chromosome. It should be emphasized that the majority of NOR-bearing chromosomes were associated with nucleoli and the number of nucleoli-associated chromosomes generally exceeded the number of competent NORs (Fig. 3a; Smirnov et al., 2006).

In HeLa cells, chromosome 15, being the main contributor of competent NORs (Smirnov et al., 2006), was at the same time the most frequently associated with nucleoli (Fig. 3). Chromosomes 13 and 14, regularly containing, respectively, one and three NORs, but only one competent NOR (Smirnov et al., 2006), contacted nucleoli with a low frequency. Chromosomes 21 and 22 possessing an intermediate number of competent NORs (Smirnov et al., 2006) showed also an intermediate level of nucleolar association.

To assess correlation between the number of transcriptionally competent NORs in particular chromosomes and their association with nucleolus in LEP cells, we chose to compare chromosome 15 and 13, because they showed the most regular pattern and represented correspondingly the maximum and minimum number of competent NORs (Smirnov et al., 2006). We found that chromosomes 15 associated with nucleoli by far more frequently than chromosomes 13 (Fig. 3). This indicated that in the nuclei of diploid LEP cells, chromosomes carrying competent NORs had a tendency to be associated with nucleoli similarly as in the transformed HeLa cells.

We thus conclude that the tendency of rDNA-bearing chromosome homologs to associate with nucleoli correlates with the number of transcriptionally competent NORs in these homologs.

### 3.2 The majority of the transcriptionally non-competent NORs in HeLa and LEP cells are situated within the nucleoli

**Fig. 4:**
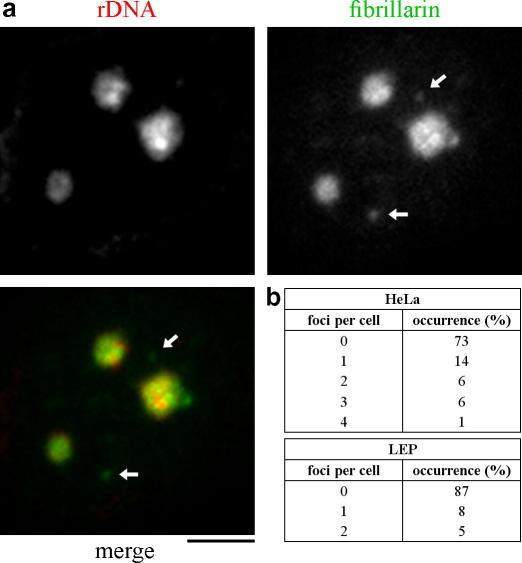
Most HeLa and LEP cells contain no extranucleolar rDNA. (a) rDNA (red), fibrillarin (green) and merged image in a HeLa cell. No rDNA signals are present outside the fibrillarin-positive nucleoli. The arrows indicate Cajal bodies. Bar: 10 μm. (b) The percentage of extranucleolar rDNA foci in HeLa and LEP cells. The extranucleolar rDNA foci are absent in about 73% of HeLa cells and in about 87% of LEP cells.

A simple explanation of the observed regularity in the nucleolar associations of the NOR-bearing chromosomes can be provided by supposing that only transcriptionally active or competent NORs participate in the formation of nucleoli. To check this hypothesis, we combined *in situ* hybridization with rDNA probe and fibrillarin immunolabeling (Fig. 4a). Although for obvious reasons we could not count individual NORs within the nucleoli, this procedure enabled us to count rDNA foci localized outside the fibrillarin-positive nucleoli (Fig. 4b). Of course, such extranucleolar foci could include two or more coalesced rDNA clusters. Despite this, we found that the extranucleolar rDNA foci were absent in about 73% of HeLa cells and about 87% of LEP cells (Fig. 4b). We can, therefore, conclude that most NORs, both competent and non-competent, should be situated in nucleoli.

**Fig. 5:**
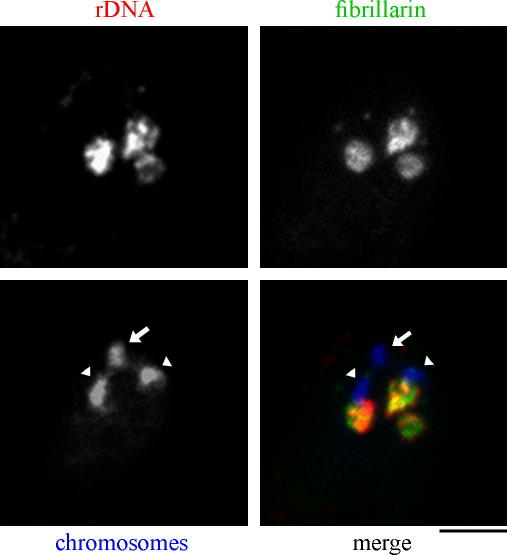
Triple labeling of chromosomes 14, fibrillarin and rDNA in a HeLa cell. The chromosomes are shown in blue, fibrillarin in green, and rDNA in red. Two chromosome territories are closely associated with nucleoli (arrowheads). The third chromosome territory (arrow) is distanced from the nucleolus and is not colocalized with rDNA signal. Since no rDNA signals were found outside the nucleoli, the NOR belonging to this chromosome should be in the nucleolus. Bar: 10 μm.

Since some chromosomes are quite frequently not associated with nucleoli (Figs. 1 and 3a), and most of non-competent NORs are found in nucleoli, our results also imply that some NORs may be distanced from the respective chromosome territories. To confirm this, we used the fact that all three homologs 14 in HeLa cells carry NORs (Smirnov et al., 2006), and not all of these chromosomes are found in association with nucleoli (Figs. 2 and 3a). Performing FISH experiment with probes for chromosome 14 and for rDNA, in combination with fibrillarin immunolabeling, we regularly found that some extranucleolar chromosome territories were not co-localized with any rDNA (Fig. 5). Therefore, some NORs, or at least their parts containing rDNA coding regions, should be located within nucleoli, and connected to the respective chromosome territories via thin filamentous protrusions. Indeed, we sometimes identified a weak FISH signal connecting the chromosome 14 territory with the nucleolus (Fig. 2). Since the nucleolar association of NOR-bearing homologs correlates with the number of transcriptionally competent NORs in these homologs, we speculate that the NORs distanced from their chromosome territories are non-competent.

We thus conclude that most of the transcriptionally non-competent NORs are situated in the nucleoli, and some NOR-bearing chromosomes are positioned in such manner that their NORs are located in the nucleolus, while the bulk of the chromosome territory is distanced from the nucleolus.

## 4 Discussion

Results of the present work expand our knowledge of the intricate spatial relationship among nucleoli, NOR-bearing chromosomes and both competent and non-competent NORs in the interphase nucleus of human-derived HeLa and LEP cells.

We found that the higher is the number of competent NORs in the given NOR-bearing homologs, the higher is the frequency of close nucleolar associations of these homologs (Fig. 3). It should be mentioned, however, that the given human homologs may carry predominantly either competent or non-competent NORs depending on the cell type. For instance, chromosomes 15 carry maximal number of competent NORs in both HeLa and LEP cells (Smirnov et al., 2006), whereas in human lymphocytes these chromosomes carry lower number of competent NORs than the other type of NOR-bearing homologs (Heliot et al., 2000).

The correlation between the close nucleolar associations of the NOR-bearing chromosomes and the transcription competence of their NORs, as observed in this study, can be explained straightforwardly by the effect of the rDNA transcription activity on the chromosome positioning. We have to mention in this respect that the total number of the competent NORs does not change significantly during the cell cycle (Smirnov et al., 2006), and rDNA transcription starts at the end of mitosis, when NORs still exist as individual entities. Both nucleoli and chromosome territories are formed later on in G1 phase (Gebrane-Younes et al., 1997; Raška et al., 2006; Roussel et al., 1996; Smirnov et al., 2006; Weisenberger and Scheer, 1995). On the other hand, we observe in the present study that the number of chromosomes closely associated with nucleoli generally exceeds the number of competent NORs (Fig. 3a, Smirnov et al., 2006). For instance, in the case of HeLa chromosomes 14, only one of three NORs is usually competent, while in average more than one chromosome was found in close association with nucleoli (Figs. 2 and 5). Thus, nucleolar association of the NOR-bearing chromosome is not sufficient for the activity of its NOR. These data, therefore, suggest that transcription competence of NORs represent one factor that can cause the respective chromosome association with the nucleoli, but other players are apparently involved as well. Beside the role of the transcribed sequences of ribosomal genes, of course, together with associated protein and nucleoprotein complexes (Raška et al., 2006), we cannot exclude a role of other sequences including also the non-transcribed spacers and flanking sequences that can be found within, or at the periphery, of nucleoli (Kaplan et al., 1993; Santoro, 2005).

Our data enable us to draw an interesting conclusion about the localization of non-competent NORs. On the one hand, most of the HeLa and LEP cells do not possess any extranucleolar rDNA. On the other hand, all HeLa cells and more than 95% of LEP cells contain at least one non-competent NOR during mitosis (Smirnov, unpublished observations). Moreover, after treatment with calyculin A, that allows visualization and quantification of individual NORs in interphase, we find the same number of non-competent NORs as in mitosis (Smirnov et al., 2006). Therefore we conclude that a vast majority of NORs must be localized in nucleoli.

Nucleolar location of the non-competent or transcriptionally silent NORs has been reported in several other studies. For instance, in a mouse/human hybrid system, where mouse cells contained one human acrocentric chromosome (Sullivan et al., 2001), the NOR belonging to this chromosome was localized in nucleolus, but remained transcriptionally silent. Clusters of inactive, methylated rDNA repeats were found in the nucleoli of normal mouse neurons (Akhmanova et al., 2000), although it was not demonstrated that these clusters consisted of silent NORs. Importantly, here we present the first focused documentation testifying for the accommodation of the majority of non-competent, and so presumably silent, NORs in the nucleoli of human cells. It is not yet clear why non-competent NORs are situated in the nucleolus, and we are unable to further discuss the causes for this phenomenon on the basis of results of the present study. Our data, however, indicate that the localization of NORs in the nucleolus is to some extent independent from their transcription activity.

Using triple labelings of the nucleoli, rDNA and chromosome 14 territory (Fig. 5), we have shown that some NORs (supposedly non-competent) are situated as far as several micrometers away from the areas occupied by the corresponding chromosome territory. In such cases we sometimes observe extended protrusions of chromatin connecting extranucleolar chromosome territories with the nucleoli. An analogous situation has been also described for a number of non-ribosomal genes that are found to be located on the long loops extending from the chromosome territories (Bártová et al., 2007; Baxter et al., 2002; Chubb and Bickmore, 2003; Mahy et al., 2002; Kioussis, 2005; Morey et al., 2007).

In summary, the large-scale positioning of the NOR-bearing chromosomes, through their associations with nucleoli, is closely linked to the transcription activity of rDNA. The tendency of rDNA-bearing chromosomes to associate with nucleoli correlates with the number of transcriptionally competent NORs in the respective chromosome homologs. Importantly, not only competent, but also most of non-competent NORs, are included in the nucleoli. Some intranucleolar NORs are situated on elongated chromatin protrusions connecting nucleoli with respective chromosome territories distanced from nucleoli.
